# Triterpenoids from *Aglaia abbreviata* exert cytotoxicity and multidrug resistant reversal effect in MCF-7/ADM cells via reactive oxygen species induction and P-glycoprotein inhibition

**DOI:** 10.18632/oncotarget.17287

**Published:** 2017-04-20

**Authors:** Juan Cen, Beibei Zheng, Rubing Bai, Li Zhang, Feng Zhang, Xia Zhang

**Affiliations:** ^1^ College of Pharmacy, Henan University, Kaifeng, People’s Republic of China; ^2^ Key Laboratory of Natural Medicine and Immune Engineering, Henan University, Kaifeng, People’s Republic of China; ^3^ State Key Laboratory of Biotherapy and Cancer Center, West China Hospital, Sichuan University, and Collaborative Innovation Center for Biotherapy, Chengdu, People’s Republic of China

**Keywords:** multidrug resistance, reactive oxygen species, Aglaia abbreviata, NF-E2-related factor 2, P-glycoprotein

## Abstract

Triterpenoids from the *Aglaia* have been shown cytotoxicity on a broad spectrum of human tumor cells. In the present study, we extracted triterpenoids AA-5 (1) and AA-6 (2) from stems of *Aglaia abbreviata*, and studied their cytotoxicity in multidrug resistant (MDR) MCF-7/ADM cells. After 48 h treatment, AA-5 (1) and AA-6 (2) significantly increased mitochondrial-mediated apoptosis by enhancing reactive oxygen species (ROS) with depressed mitochondrial membrane potential and caspase-9 activities. The drug efflux transporter P-glycoprotein (P-gp) and the intracellular antioxidant systems, involving Glutathione S-Transferase π, Glutathione and heme oxygenase-1, were also inhibited via the ROS-depressed Akt/NF-E2-related factor 2 pathway. Furthermore, 2 h-treatment of AA-6 (2) at non-toxic concentrations exhibited MDR reversal effects with no alteration on P-gp expression but increased drug accumulation ability. AA-6 alos demonstrated synergetic effects with classic anti-tumor agents. Moreover, computational modeling studies showed that AA-6 (2) might bind to the modulator site on P-gp and act as an inhibitor, not a substrate of P-gp. Therefore, AA-5 (1) and AA-6 (2) may be effective anti-tumor and reversal agents for the further development of therapeutics against MDR breast cancer.

## INTRODUCTION

Multidrug resistance (MDR), one of the most serious obstacles in cancer chemotherapy, makes malignancy cancer cells respond insufficiently to a spectrum of structurally and functionally unrelated anticancer agents. Mechanisms with cross-talk responsible for MDR include over-expressed efflux transporters, over-activated detoxification systems and imbalanced apoptosis/proliferation [1, 2]. P-glycoprotein (P-gp) is one of the well-studied transmembrane glycoproteins involved in MDR, which is overexpressed in various MDR cell lines and functions as an ATP-dependent drug efflux pump [3]. P-gp-mediated MDR has also been associated with the inhibition of multiple forms of caspase-dependent tumor cell apoptosis [4]. Because single P-gp inhibitors showed limited clinical activity in clinical study [5] and drug combination usually led to cumulative toxicities and unpredictable drug-drug interactions, apoptosis inducers with multiple targets appear to be attractive to achieve better effects in chemotherapy.

Effective tumor treatment usually requires the use of toxic chemotherapy, which mainly lead to the massive generation of reactive oxygen species (ROS) [6, 7]. However, the MDR will be generated with the increased cellular adaptation to oxidants by inducing antioxidants and detoxification molecules [8]. NF-E2-related factor 2 (Nrf2) is one of the most important cellular defense mechanisms against oxidative stress. Nrf2 mediates anti-oxidative response as a transcription factor. And Nrf2-activated cytoprotective genes include heme oxygenase-1 (HO-1), glutathione S-transferase (GST), and P-gp [9, 10]. Therefore, constitutive Nrf2 activation is recognized to be responsible for chemoresistance in tumors [8]. The rectification of the overactivated intracellular antioxidants and detoxification system is a promising way to sensitize chemotherapy in chemo-resistant cancer.

**Figure 1:**
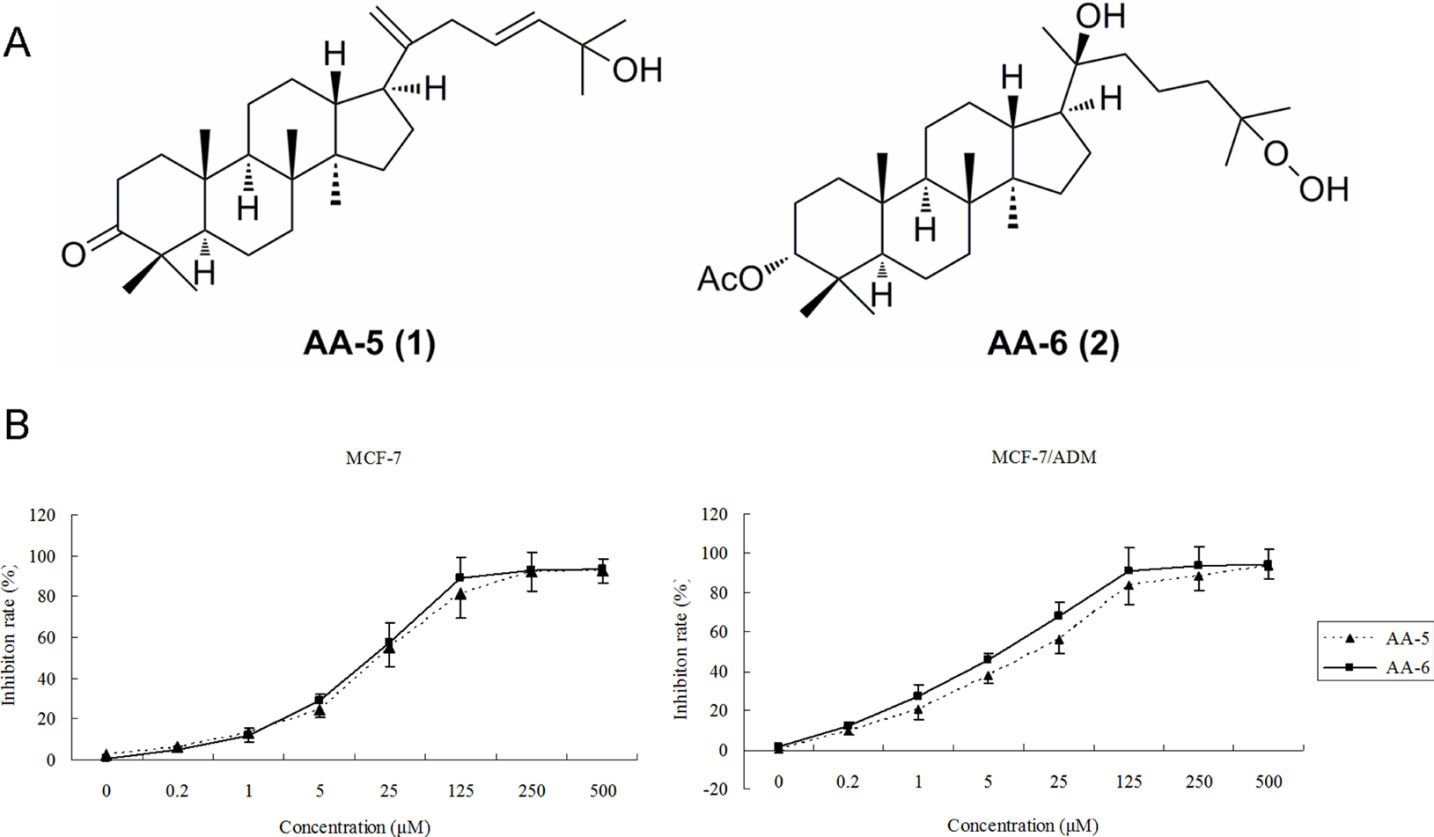
Inhibition of cell viability by AA-5 (1) and AA-6 (2) (**A**) The structure of AA-5 (1) and AA-6 (2). (**B**) AA-5 (1) and AA-6 (2) inhibited growth of MCF-7 and MCF-7/ADM cells in a dose-dependent manner by MTT assay. Cells were treated with same volume of DMSO, 0.2, 1, 5, 25, 125, 250, 500 µM AA-5 (1), AA-6 (2) on MCF-7 and MCF-7/ADM cells for 48 h. Data represents the mean ± SD, *n* = 3.

Many studies have revealed anti-tumor effects of triterpenoids from *Aglaia* on a broad spectrum of human tumor cells [11–14]. Our previous study reported that two dammarane-type triterpenoids Aglaiabbreviatin E and F (AA-5 (1) and AA-6 (2), AAs, Figure 1A), isolated from the stems of *Aglaia abbreviata*, demonstrated great cytotoxicity not only in sensitive tumor cell lines but also in their drug resistant counterparts [15]. The present work was a detailed mechanism study of their activities in MDR human breast cells, demonstrating that AAs may serve as prospective anticancer agents via induction of ROS and inhibition of P-gp.

## RESULTS

### Inhibitory effects on MCF-7 and MCF-7/ADM cells

As shown in Figure 1B, AA-5 (1) and AA-6 (2) inhibited the viability of MCF-7 and MCF-7/ADM cells in a dose-dependent manner. After the treatments for 48 h, the 50% inhibition concentrations (IC_50_) of AA-5 (1) by MTT assay are 14.80 µM and 10.22 µM in MCF-7 and MCF-7/ADM cells, respectively, while those of AA-6 (2) are 12.46 µM and 6.1 µM. Notably, the inhibitory effects of AA-5 (1) and AA-6 (2) were much stronger in MDR cells than those in sensitive cells. Importantly, AA-6 (2) were more effective than that of adriamycin (IC_50_ = 57.3 µM) and vincristine (IC_50_ = 7.5 µM) in MCF-7/ADM cells.

### Enhanced mitochondrial apoptotic pathway

**Figure 2:**
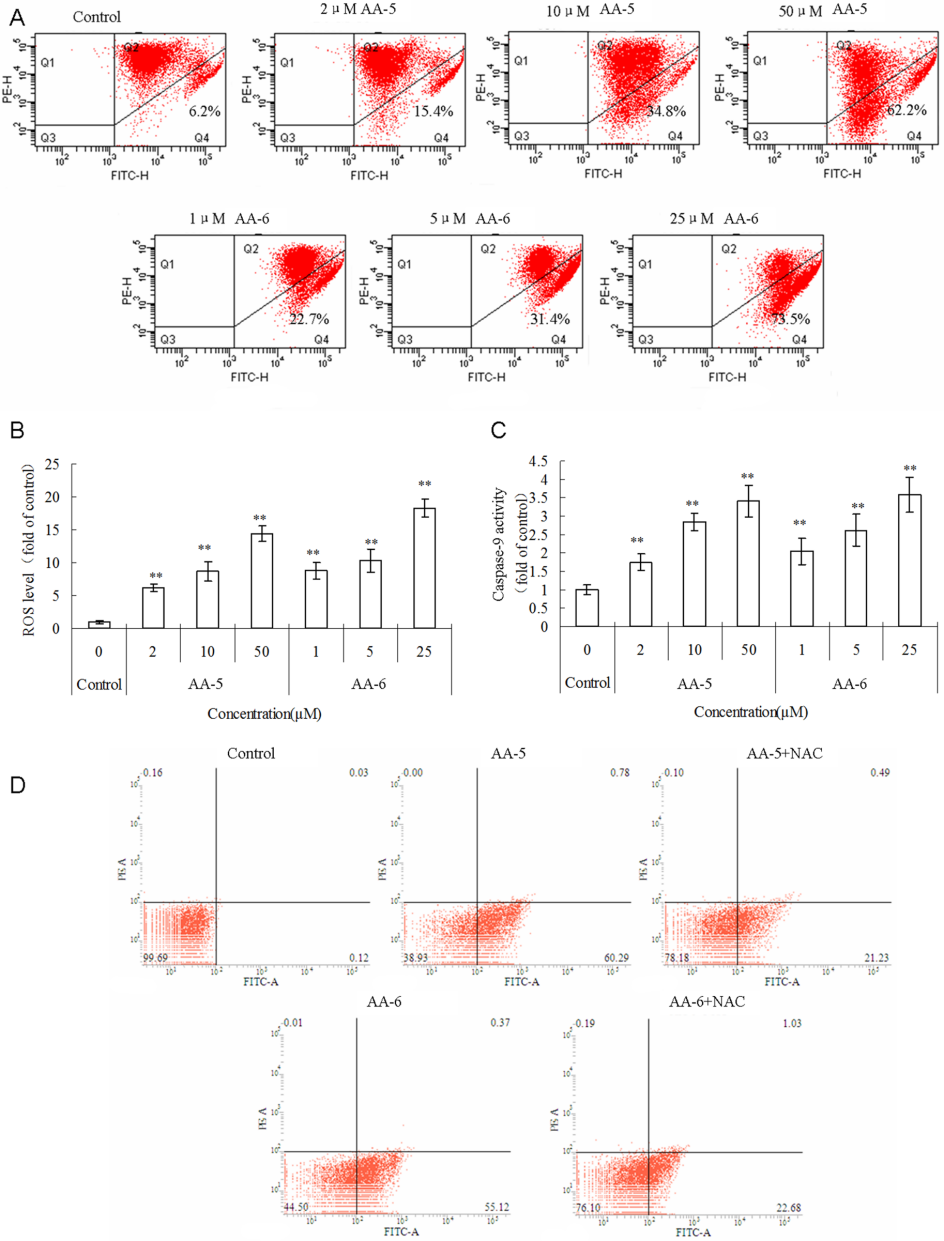
AA-5 (1) and AA-6 (2) induced mitochondria-mediated apoptosis in MCF-7/ADM cells (**A**) Concentration-dependent depolarization of mitochondria membrane potential in response to 2, 10, 50 µM AA-5 (1) and 1, 5, 25 µM AA-6 (2) treatment measured by flow cytometry. (**B**) Concentration-dependent increase of reactive oxygen species generation in response to 2, 10, 50 µM AA-5 (1) and 1, 5, 25 µM AA-6 (2) treatment. (**C**) Activation of caspase-9 induced by 2, 10, 50 µM AA-5 (1) and 1, 5, 25 µM AA-6 (2) treatment. Data represents the mean ± SD, *n* = 3, significant differences relative to control were indicated as **P < 0.01. (**D**) Flow cytometric analysis of AA-5 (1) and AA-6 (2) induced apoptosis in MCF-7/ADM cells. Cells were incubated with 10 µM AA-5 (1), or 5 µM AA-6 (2) with or without 10 mM NAC for 48 h. Cells in control group were treated with same volume of DMSO.

As shown in Figure 2A, cell percentage with low mitochondrial membrane potential (MMP) significantly increased in the presence of AA-5 (1) and AA-6 (2) with a concentration-dependent manner. The levels of intracellular ROS (iROS) in MCF-7/ADM cells received AA-5 (1) and AA-6 (2) treatments were measured using DCFH-DA method and shown in Figure 2B. Both AA-5 (1) and AA-6 (2) treatments caused concentration-dependent enhancement of iROS compared to the control. Because in the downstream of decreased MMP and increased ROS, caspases can be activated to cleave enzymes and proteins and lead to further progressing of apoptosis, the activity of caspase-9, a typical indicator of mitochondrial apoptotic pathway was also analyzed. As shown in Figure 2C, AA-5 (1) and AA-6 (2) both significantly elevated the activity of caspase-9 in a dose-dependent manner. Accordingly, the percentages of early apoptotic cells (Annexin V^+^/PI^−^) significantly increased to 59.1 ± 3.9% and 53.8 ± 3.6% after the treatments with 10 µM AA-5 (1), or 5 µM AA-6 (2) for 48 h (Figure 2D), while the percentage in the control group was 0.7 ± 0.5%. Moreover, 10 mM ROS scavenger NAC significantly restored the cell percentages of early apoptosis in the presence of 10 µM AA-5 (1) or 5 µM AA-6 (2) to 24.6 ± 3.9% and 24.0 ± 3.4%, respectively, suggesting that the apoptosis inductions of AA-5 (1) and AA-6 (2) were in ROS-dependent manner.

### Decreased P-gp and intracellular antioxidants via ROS-inhibited Akt/Nrf2 pathway

**Figure 3:**
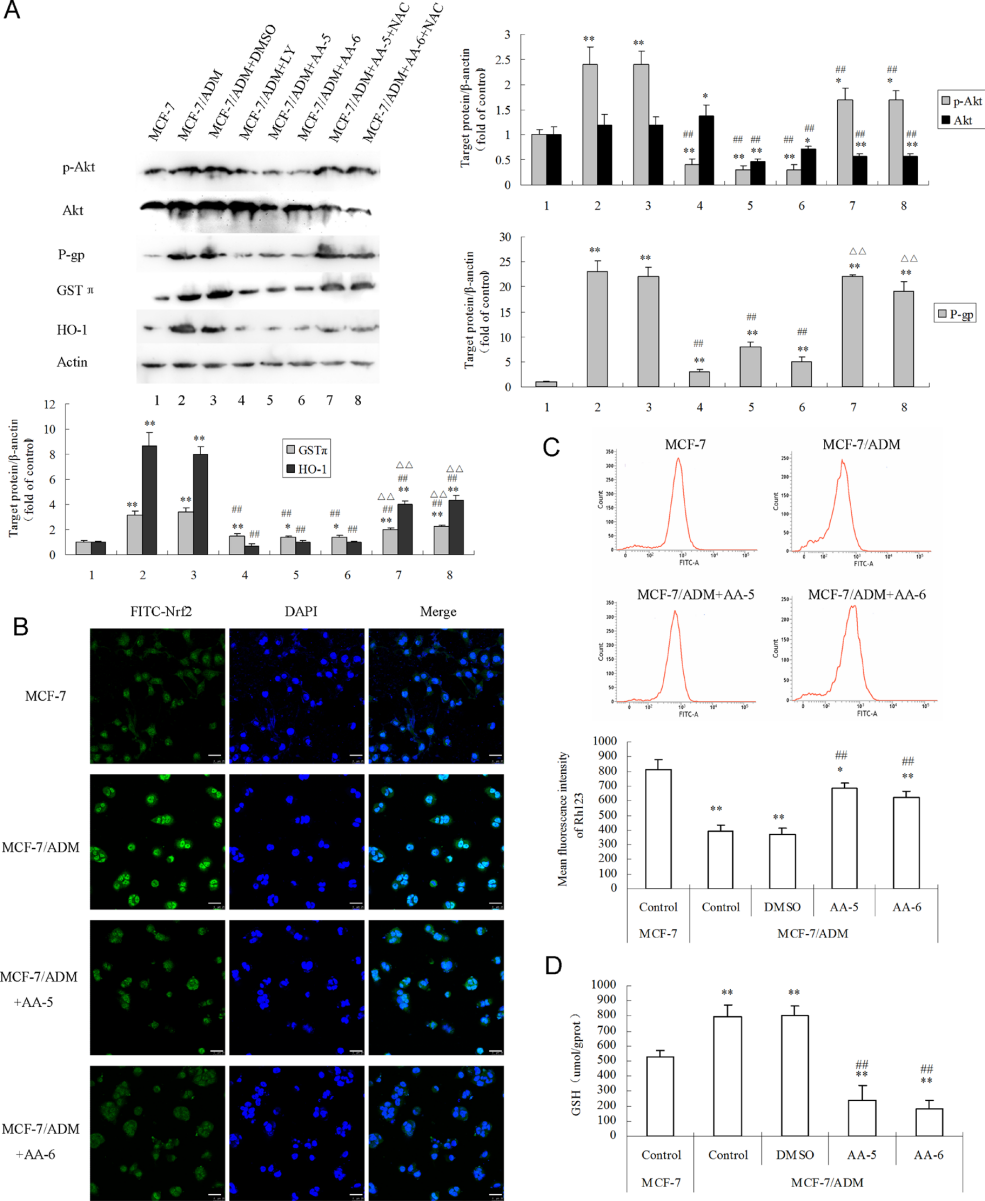
AA-5 (1) and AA-6 (2) inhibited P-gp and cellular anti-oxidant ability in MCF-7/ADM cells via ROS/Akt and Nrf2 signal pathways (**A**) AA-5 (1) and AA-6 (2) inhibited P-gp, GSTπ, HO-1 expression in MCF-7/ADM cells. Cells were respectively treated with same volume of DMSO, 10 μM LY294002, 10 µM AA-5 (1), 5 µM AA-6 (2), 10 µM AA-5 (1) + 10 mM NAC, and 5 µM AA-6 (2) + 10 mM NAC for 48 h. (**B**) Immunohistochemical analysis of Nrf2 nuclear translocation. Cells were treated with or without 10 µM AA-5 (1), 5 µM AA-6 (2) for 48 h. The length of the white bar in Figure 3B is 25 μm. (**C**) Effects of AA-5 (1) and AA-6 (2) on accumulation of Rhodamine 123 (Rh123) in MCF-7/ADM cells. MCF-7/ADM cells were firstly incubated with same volume of DMSO, 10 µM AA-5 (1), or 5µM AA-6 (2) for 48 h, and then 5 µM Rh123 for 1 h. (**D**) Effects of AA-5 (1) and AA-6 (2) on GSH contents in MCF-7/ADM cells. MCF-7/ADM and MCF-7 cells were treated with or without 10 µM AA-5 (1), 5 µM AA-6 (2) for 48 h. Data represents the mean ± SD, *n* = 3, significant differences relative to MCF-7 cells were indicated as ^**^P < 0.01, significant differences relative to MCF-7/ADM cells were indicated as ^##^*P* < 0.01.

As shown in Figure 3A, MCF-7/ADM cells had much higher protein levels of p-Akt, P-gp, GSTπ, and HO-1 than MCF-7 cells. Treatments of 10 µM AA-5 and 5 µM AA-6 for 48 h both significantly reduced expressions of p-Akt, P-gp, GSTπ, and HO-1 in MCF-7/ADM cells. The PI3K inhibitor LY294002 showed similar effects on these proteins as well. Co-treatment with ROS scavenger NAC greatly restored the depressed p-Akt by AA-5 and AA-6, suggesting that AA-5 and AA-6-mediated depression of Akt pathway was in a ROS-dependent way.

Since AA-5 and AA-6 significantly inhibited the expression of GSTπ and HO-1, which were typical downstream targets of Nrf2, the effects of AA-5 and AA-6 on the expression and the distribution of Nrf2 in MCF-7/ADM cells were further investigated. Results shown in Figure 3B demonstrated that MCF-7/ADM cells had much higher levels of Nrf2 expression and nuclear translocation compared with MCF-7 cells. AA-5 and AA-6 both significantly reversed the enhancement of the expression and the translocation of Nrf2 in MDR cells. The results were in consistence with the decreased expression of P-gp, GSTπ, and HO-1.

To further evaluate the ability of altering drug efflux, the effects of AA-5 (1) and AA-6 (2) on intracellular P-gp substrate Rh123 accumulation in MCF-7/ADM cells were analyzed by flow cytometry with Rh123 (Figure 3C). The enhanced drug accumulation by AA-5 (1) and AA-6 (2) was in accordance with the decreased P-gp expression. To further assess the intracellular antioxidant ability, the level of antioxidant GSH was also monitored in the presence of AA-5 (1) and AA-6 (2). As shown in Figure 3D, MCF-7/ADM cells had a much higher level of GSH content than MCF-7 cells, while AA-5 (1) and AA-6 (2) significantly decreased the level of GSH in MCF-7/ADM cells.

### The effect of short-term treatment

**Figure 4:**
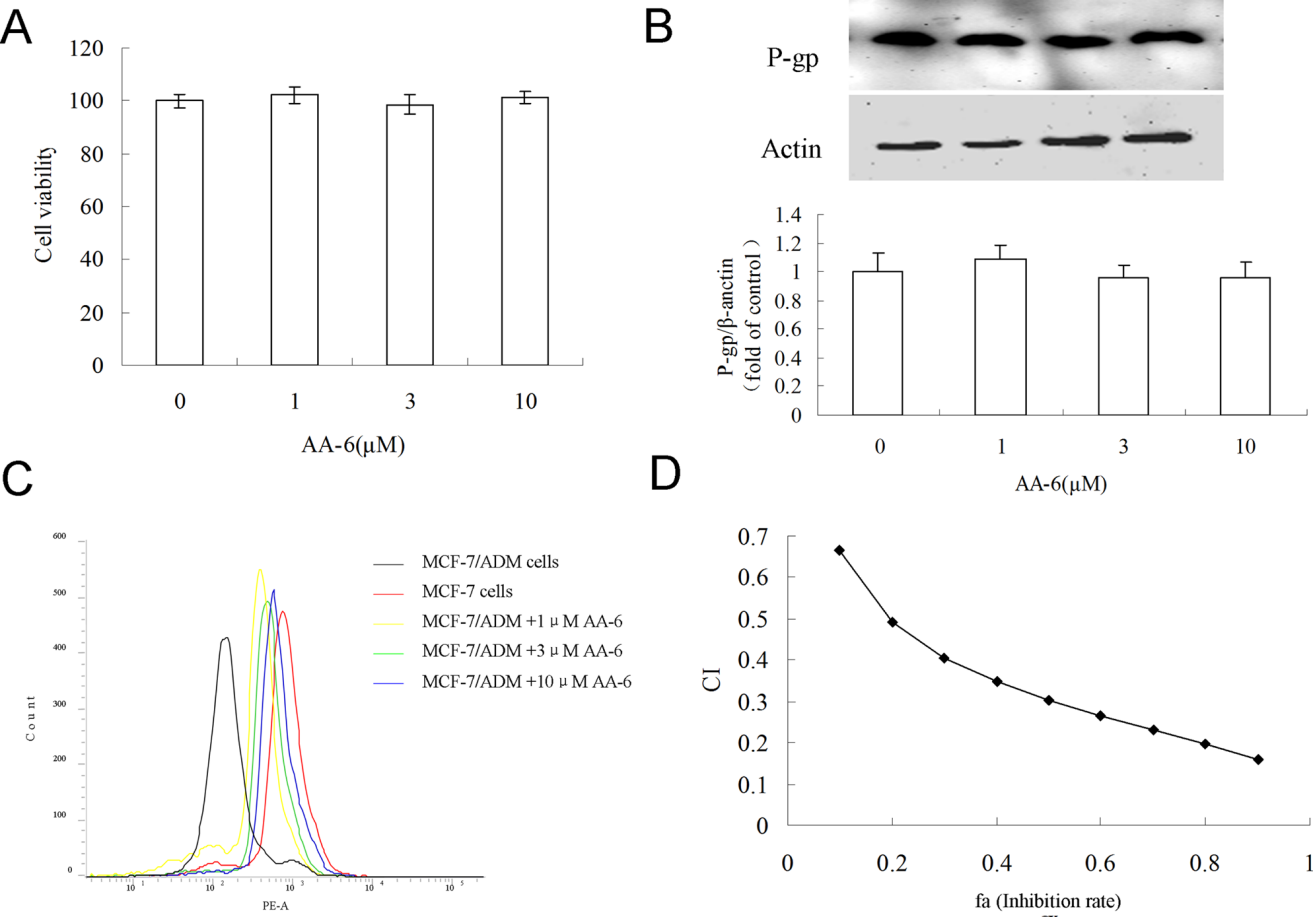
The short-term effect and the synergistic effect of AA-6 (2) on MCF-7/ADM cells (**A**) Treatment of same volume of DMSO, 1, 3, 10 µM AA-6 (2) for 2 h showed no cytotoxicity in MCF-7/ADM cells. Data represents the mean ± SD, *n* = 3. (**B**) Treatment of same volume of DMSO, 1, 3, 10 µM AA-6 (2) for 2 h had no significant effect on expression of P-gp. (C) AA-6 (2) concentration-dependently increased the accumulation of Rhodamine 123 (Rh123) in MCF-7/ADM cells. MCF-7/ADM cells were firstly incubated with same volume of DMSO, 1, 3, 10 µM AA-6 (2) for 2 h, and then 5 µM Rh123 for 1 h. Cells were collected for the determination of intracellular Rh123. (D) CI plots for analysis of the combination of AA-6 (2) with adriamycin in MCF-7/ADM cells. CI = 1, < 1, and > 1 indicates additive effect, synergism, and antagonism, respectively.

Given the remarkable effects of AA-6 (2) on P-gp expression and drug accumulation ability in the 48h-treatment, short-term treatment (2h) were utilized to further investigate the capability of AA-6 (2) as a MDR reversal agent. Concentrations of AA-6 (2) showing no cell toxicity in the 2h-treatment were chosen in this experiment (Figure 4A). Figure 4B and 4C showed that AA-6 (2) treatments at 1, 3, and 10 µM for 2 h did not alter the expression of P-gp in MCF-7/ADM cells, but significantly increased the intracellular accumulation of P-gp substrate Rh123 in a concentration-dependent manner. The results suggested AA-6 (2) might act as a MDR sensitizer in chemotherapy. Therefore, the synergetic effects of AA-6 (2) with classic anti-tumor agent adriamycin were further analyzed. The results in Figure 4D illustrated that there was a strong synergy between AA-6 (2) and adriamycin for drug combinations in MCF-7/ADM cells (CI < 1). The other indexes of Dm, m, and r were all shown in Table 1.

**Table 1:** Concentration-effect relationships of AA-6, alone and in combination with adriamycin (ADM) in MCF-7/ADM cells

Drug	Concentration	Inhibition rate	Parameters*		
	(μM)	(fa)	Dm	m	*r*
ADM	1	0.013	36.08	1.085	0.959
	3	0.094			
	10	0.275			
	30	0.392			
	100	0.724			
AA-6	1	0.277	5.26	0.673	0.979
	3	0.384			
	10	0.565			
	30	0.746			
	100	0.904			
ADM&AA-6	2	0.464	2.70	0.959	0.987
	6	0.622			
	20	0.893			
	60	0.944			
	200	0.985			

^*^Dm, median effect dose (concentration in micromoles/liter that inhibits cell growth by 50%); m, shape of the dose-effect curve (m = 1, hyperbolic; m > 1, sigmoidal; m < 1, negative sigmoidal); *r*, linear correlation coefficient of the median effect plot.

### Computational modeling studies

**Figure 5:**
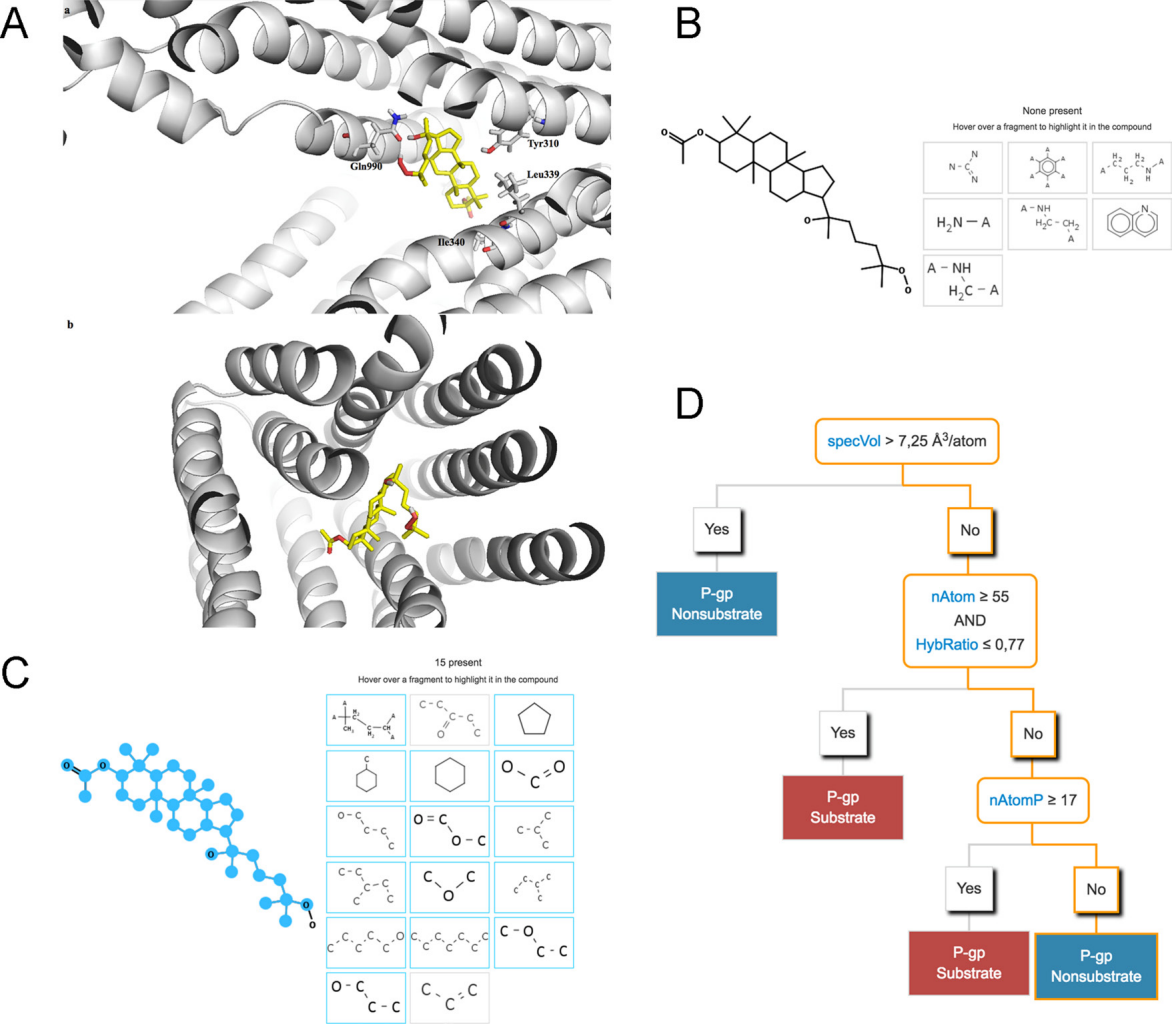
AA-6 (2) may bind with P-gp and act as an inhibitor, not a substrate of P-gp (**A**) The predicted binding configuration of AA-6 (2) (yellow, nonpolar hydrogens undisplayed) into the modulator binding site on P-gp (shown in grey). (a) a side view of the predicted binding configuration of AA-6 (2); (b) a bottom view of the predicted binding configuration of AA-6 (2). (**B**) None of the effluxophore fragments (shown in the boxes) present in AA-6 (2). (**C**) Fifteen of the seventeen anti-effluxophore fragments (shown in the boxes) present in AA-6 (2). (**D**) The decision tree model and its prediction of AA-6 (2) (the decision flow shown in orange outlines).

We performed the docking study of AA-6 (2) into the homology model of human P-gp using GOLD (version 5.0 [16]) to investigate the potential interactions of AA-6 (2) and P-gp. The predicted binding mode of AA-6 (2) in human P-gp was illustrated in Figure 5A. AA-6 (2) was docked at the top of the cavity located next to the outer leaflet of the lipid bilayer. This cavity is the modulator QZ59-RRR binding site, which is also referred as the M-site by dos Santos and co-workers [17]. In general, AA-6 (2) fitted well with the M-site, indicating that AA-6 (2) might inhibit P-gp through binding to the modulator binding site. The hydroxyl group and hydroperoxyl group of AA-6 (2) hydrogen-bonded with the backbone carbonyl group of Gln990. The hydrophobic moiety of AA-6 (2) formed extensive hydrophobic interactions with residues Leu65, Met69, Phe303, Ile306, Try307, Tyr310, Phe335, Leu339, Ile340, Phe343, Gly346, Phe383, Phe728, Tyr953, Phe978, Val982, Met986, and Ala987.

To further investigate whether AA-6 (2) binds to P-gp as a substrate, we applied Supek’s computational models. Supek’s support vector machine (SVM) classification model was trained on a large set of 814 known P-gp substrates and non-substrates [18]. It is one of the most accurate P-gp substrate prediction models and achieved high accuracy of 86.7% on a separate testing set. Based on this model, AA-6 (2) was predicted to be a non-substrate of P-gp with high confidence (*P* = 0.95). It did not contain any of the seven molecular fragments (effluxophores) that frequently appeared in P-gp substrates as shown in Figure 5B. It instead contained fifteen of the seventeen molecular fragments (anti-effluxophores) that frequently appeared in P-gp non-substrates (Figure 5C). Based on Supek’s decision tree model, AA-6 (2) was also predicted to be a non-substrate of P-gp. The normalized molecular van der Waals volume (specVol) of AA-6 (2) is 5.96 7.3 Å^3^/atom. The number of atoms (nAtom) of AA-6 (2) is 90. The Hybridization ratio (HybRatio, nsp3/(nsp3 + nsp2)) of AA-6 (2) is 0.97. The number of atoms in the largest π chain (nAtomP) of AA-6 (2) is 3.0. Thus, according to the decision tree rules, AA-6 (2) is a non-substrate of P-gp as shown in Figure 5D. In all, these computational studies showed that AA-6 (2) was likely to bind to P-gp, and act as an inhibitor and not a substrate of P-gp.

## DISCUSSION

MDR, usually resulting from a combination of mechanisms such as blocked apoptosis and increased efflux, is one of the major obstacles to the successful cancer chemotherapy [1]. Our study revealed that AA-5 and AA-6 significantly induced ROS generation and inhibited P-gp, leading to great cytotoxicity in MCF-7/ADM cells. Moreover, AAs showed stronger inhibition against tumor cells than normal dysplastic MCF-10A cells (Supplementary Figure 1). These results, especially those of AA-6, may benefit further research on *Aglaia abbreviata* and facilitate the development of novel chemotherapeutic drugs against MDR tumors.

The core mechanism of AAs-induced cell damage was via ROS generation. Firstly, AAs-induced massive ROS generation broke the redox balance and activated the process of mitochondrial apoptosis, which was characterized by depolarization of MMP and activation of caspase-9 in MCF-7/ADM cells (Figure 2) as well as in MCF-7 cells (Supplementary Figure 2). Nevertheless, it is still unknown whether external pathways participate in the AAs-induced apoptosis. Secondly, massive ROS generation led to the decrease of the expression and function of P-gp by long-term treatment of AAs (Figure 3A). Thirdly, AAs-generated ROS depressed the expression of intracellular antioxidant enzymes GSTπ and HO-1, as well as the content of antioxidant GSH (Figure 3). Because low propensity to apoptosis, over-expressed drug efflux transporters, and over-activated antioxidant system are all important mechanisms of MDR, AAs-induced cytotoxicity should mainly be the results of AAs-induced ROS generation and ROS-cascaded depression of Akt/Nrf2 signal pathways.

High level of ROS was usually proposed as the cell-damaging factors of chemotherapy [19], while sustained and moderate production of ROS in MDR cells exhibited signal effects to activate survival signaling pathways such as the PI3K/Akt pathway, which facilitates oncogenic phenotype of cancer [20, 21]. Consistently, our study demonstrated higher levels of basal intracellular ROS and activities of the Akt pathway in MDR cells than their sensitive counterparts ([10] and Figure 3A). Treatments of AA-5 (1) and AA-6 (2) for 48 h significantly increased the level of ROS (Figure 2B), and co-treatment with NAC greatly restored the AAs-decreased p-Akt expression (Figure 3A). Therefore, we speculated that AAs-generated ROS led to the decrease of p-Akt expression. Moreover, our results showed that the expressions of P-gp, GSTπ, and HO-1 were all decreased by the treatment of the Akt inhibitor LY294002 (Figure 3), suggesting P-gp, GSTπ and HO-1 was at least partially increased by Akt pathway. As is well-known, P-gp is one of the most important drug efflux transporters; GSTπ is a multifunctional enzyme that plays a critical role in cellular detoxification and drug efflux [22]; HO-1 is an inducible phase II enzyme with antioxidant activity representing an adaptive response that increases cell resistance to oxidative injury [23]. Therefore, the expressions of P-gp, GSTπ, and HO-1 decreased by AAs finally led to the inhibitions of cellular detoxification, which facilitated cytotoxicity in MCF-7/ADM cells. These effects were mediated by the depressed Akt signal, which was also the result of massive ROS generation by long-term treatment of AAs. However, the short term treatment of AAs (2 h) showed a different effect on Akt signal, in which the expression of p-Akt was increased while the level of Akt was decreased (Supplementary Figure 3). As is known, ROS can induce phosphorylation of various serine/threonine kinases, and Akt (or protein kinase B) is one of such kinases [24]. Therefore, we speculate that the short-term incubation of AAs might only generate moderate level of ROS, which activated the Akt signal pathway and stimulated cell survival [25], but had no benefit for its anti-tumor activity.

Moreover, the expression and nuclear translocation of Nrf2 were further analyzed by immunofluorescence, because GST and HO-1 are typical targets of Nrf2 [26], and PI3K/Akt signals can activate the Nrf2-mediated antioxidant response [27]. Nrf2 is a transcriptional factor of efflux transporter and a key switch-on mechanism of the upregulation of endogenous antioxidant enzymes and detoxifying systems. Therefore, it is closely associated with MDR in tumor [27, 28]. Notably, our results (see Figure 3B) showed Nrf2 was elevated in the expression and translocations to the nuclei of MCF-7/ADM cells, suggesting higher activity of Nrf2 in MDR cells than that in sensitive cells. AA-5 (1) and AA-6 (2) decreased the expression and nuclear translocations of Nrf2. Thus, we speculate that AAs-depressed expressions of GSTπ and HO-1 were mediated by Nrf2 signals and its upstream PI3K/Akt pathway. Moreover, Nrf2 is one of important targets of ROS [27]. Thus, all these alterations may be cascaded by AAs-generated ROS in MCF-7/ADM cells. Although researches showed contradictory results on the relevance between Nrf2 and P-gp [28, 29], our results demonstrated that P-gp may be a target of Nrf2 pathway in MCF-7/ADM cells. In summary, AAs-induced Akt inhibition by ROS generation may lead to the depression of Nrf2, which resulted in the deceases of P-gp, GSTπ and HO-1 and led to the final selective anticancer effects in MCF-7/ADM cells.

In addition to the P-gp inhibitory effects of the treatment of AA-6 for 48 h, the short-term incubation of AA-6 also impaired the P-gp functions in MCF-7/ADM cells. These short-term effects may be attributed to the fact that AA might bind to P-gp and act as an inhibitor of P-gp. The treatment of AA-6 for 2 h below 10 µM showed no cytotoxicity and no effects on the expression of P-gp in MCF-7/ADM cells, but it significantly increased the intracellular accumulation of P-gp substrate Rh123 (Figure 4). Therefore, AA-6 demonstrated inhibitory effects against the functions of P-gp. The docking study of AA-6 into the homology model of human P-gp further illustrated that AA-6 might inhibit P-gp through binding to the modulator binding site. Additional P-gp substrate predictions showed that AA-6 was not a substrate of P-gp (Figure 5). Because of its P-gp inhibitory effects, AA-6 has the potential to exert as an MDR reversal agent in chemotherapy. Furthermore, the median effect analysis showed a synergetic effect of AA-6 with classic anti-tumor agent adriamycin (Figure 4, Table 1). Moreover, AA-6 in combination with cisplatin and taxol also showed synergistic effects (Supplementary Figure 4). It suggests that AA-6 may also be used as a MDR reversal agent in a combination therapy against MDR tumors.

Taken together, our study reported two novel triterpenoids AA-5 (1) and AA-6 (2), extracted from *Aglaia abbreviata*, which had great cytotoxicity via ROS-mediated apoptosis induction, efflux transporter inhibition, and antioxidant system break in MCF-7/ADR cells. Long-term incubation of AA-6 (2) led to the decrease of P-gp expressions and functions, while short-term treatment showed the inhibition on P-gp functions. Modeling studies showed that AA-6 (2) might inhibit P-gp through binding to the modulator binding site and it was not a substrate of P-gp. These data may provide references for further studies on *Aglaia abbreviata*, and new insights into the development of novel chemotherapy against MDR breast tumors.

## MATERIALS AND METHODS

### Chemicals

The air-dried stems of *Aglaia abbreviata* were collected from Xishuangbanna, Yunnan Province, People’s Republic of China, in May 2014, and were authenticated by Professor Jing-Yun Cui, Xishuangbanna Botanical Garden, Chinese Academy of Sciences, People’s Republic of China. A voucher specimen has been deposited in the College of Pharmacy, Henan University (accession number AA201405). Briefly, the air-dried stems (20 kg) were extracted with 95% ethanol under reflux for three times. After removal of the solvent under a vacuum, the viscous concentrate was first suspended in H_2_O, and then partitioned with CHCl_3_ and EtOAc, successively. The CHCl_3_-soluble partition (265 g) was fractionated by column chromatography over D101 porous resin using gradient aqueous ethanol to give fractions A–F, combined according to TLC results. Fraction D (40 g) was chromatographed on a column of silica gel, eluted successively with a gradient of petroleum ether-ethyl acetate (20:1 to 1:2), to give seven subfractions (D1–D7). Subfraction D3 was chromatographed on a column of reversed-phase C_18_ silica gel, eluted with MeOH-H_2_O (5:5 to 9:1), to give four subfractions (D3a–D3d). Of these, subfraction D3d was separated by preparative HPLC, using MeOH-H_2_O (85:15, 10 mL/min) as the mobile phase, to give AA-5 (1) (15 mg). Subfraction D6 was chromatographed on a column of reversed-phase C_18_ silica gel, eluted with MeOH-H_2_O (5:5 to 9:1), to give five subfractions (D6a–D6e). Subfraction D6b was separated by preparative HPLC, using CH_3_OH-H_2_O (85:15, 10 mL/min) as the mobile phase, to give AA-6 (2) (20 mg). AA-5 and AA-6 were identified by comparison of their spectroscopic data with reported values (purity > 98%, as determined by HPLC-UV). Fetal bovine serum (FBS) was obtained from Zhejiang Tianhang Biotechnology (Huzhou, China). 5-Diphenyl tetrazolium bromide (MTT), 2′, 7′-dichlorodihydrofluorescein diacetate (DCFH-DA), N-Acetyl Cysteine (NAC), Rhodamine 123 (Rh123), adriamycin (ADM), and LY294002 were purchased from Sigma Chemical (St. Louis, MO, USA). Antibodies against P-glycoprotein (P-gp, sc-55510, 1:100), Nrf2 (sc-722, 1:200 for WB, 1:50 for IF), Akt (sc-8312, 1:200), p-Akt (sc-7985-R, 1:100), and HO-1 (sc-10789, 1:200) were purchased from Santa Cruz Biotechnology (CA, USA). Antibodies against glutathione S-transferase-π (GSTπ, ab53943, 1:2000) and β-actin (ab8227, 1:5000) were products of Abcam (ab, MA, USA). Horseradish peroxidase-conjugated (BA1054, BA1050, 1:2000) and FITC-conjugated secondary antibodies (BA1101, 1:50) were purchased from Boster Bio-Engineering Limited Company (Wuhan, China). Whole cell lysis buffer containing proteasome inhibitor, BCA protein kit, Annexin V-FITC Apoptosis Detection Kit, Caspase-9 Activity Assay Kit, Mitochondrial membrane potential assay kit, and Total Glutathione Assay Kit were from Beyotime Institute of Biotechnology (Nantong, China). Enhanced chemiluminescence (ECL) detection Kit was obtained from Amersham Biosciences (Buckinghamshire, UK).

### Cell culture and treatment

MCF-7 cells were from American Type Culture Collection (ATCC, Manassas, VA, USA). MCF-7/ADM cells, which were obtained from MCF-7 cells by exposure to ADM with stepwise increased concentrations, were purchased from Keygen Biotech (Nanjing, China). MCF-7 and MCF-7/ADM cell lines were respectively grown in DMEM medium and RPMI 1640 medium, containing 10% bovine serum (FBS) and 100 U antibiotics (benzylpenicillin sodium and gentamycin sulfate) at 37°C in a humidified 5% CO_2_ incubator. To maintain resistant phenotype, 1 μg/ml adriamycin was added into MCF-7/ADM cultures and maintained in drug-free medium for at least two weeks before used.

### Cell inhibition assay

The inhibition of cell viability was assessed by MTT assay. Cells of each group were harvested and seeded in 96-well plates at a density of 1 × 10^5^ cells/ml for viability assay. Then the cells were treated with same volume of DMSO, 0.2, 1, 5, 25, 125, 250, 500 µM AA-5 (1), AA-6 (2) on MCF-7 and MCF-7/ADM cells for 48 h. and then cells were incubated with MTT at 37°C for 4 h, and then the medium were removed and 150 μl DMSO were added to each well. Plates were agitated and the optical density was measured at 570 nm using a spectrophotometer (Thermo Fisher Scientific, Inc.). Inhibition rates were calculated on a plate-by-plate basis for test wells relative to control wells.

### Analysis of mitochondrial membrane potential (MMP)

The membrane potential assay is based on the JC-1 dye. Briefly, cells were separately treated various chemicals for 48 h and were incubated with 5 μg/mL JC-1 for 20 min at 37°C in the dark. The cells were rinsed twice with JC-1 staining buffer, and the fluorescence was measured by Flow Cytometry (BD FACSVerse).

### ROS assay

Cells were seeded on 6-well plates. After incubation with various chemicals, cells from each group at a density of 1 × 10^6^ were harvested and incubated with 10 μM DCFH-DA at 37°C for 30 min in the dark. Then cells were washed with PBS, and the fluorescence intensity was measured for ROS with Flow Cytometry (BD FACSVerse).

### Caspase-9 activity detection

The activity of caspase-9 was measured using the Caspase-9 Activity Assay Kit according to the product manual. Briefly, cells with or without treatment were lysed using 100 μl of lysis buffer, and subsequently centrifuged at 12 000 g for 10 min at 4°C. The supernatant was collected for the assay. Next, 40 µl of assay buffer was mixed with 50 µl of lysate in a 96-well plate, followed by the addition of 10 μl of 2 mM Ac-LEHD-pNA. The plate was incubated at 37°C in the dark for 1 h, and the relative fluorescence unit value for the emission at 405 nm was measured. The result was normalized to the total protein measured using the Bradford assay.

### Determination of apoptotic rate

The apoptotic rate was detected by Annexin V-FITC/PI double labeling method. Briefly, the cell suspension was centrifuged and re-suspended in 195 μL Annexin V-FITC binding buffer and incubated with 5 μL Annexin V-FITC in the dark at ambient temperature for 10 min. Cells were then centrifuged, and the pellet was re-suspended in 195 μL binding buffer. Cells were then incubated with 10 μL PI solution on an ice bath in the dark for 10 min. The suspension of each group was analyzed by Flow Cytometry (BD FACSVerse).

### Western blot

Cells from each group were treated with the agents for 48 h or 2 h and suspended in lysis buffer for 30 min with shaking at 4°C. After centrifugation (12 000 × g) for 10 mins, the supernatants were collected. Cell lysate (50 μg) was resolved on 4–12 % SDS-PAGE gels and then transferred on to nitrocellulose membranes. The membranes were blocked with Tris-buffered saline with 0.1% Tween 20 and 5% skim milk, and then incubated with primary antibodies overnight at 4°C. Then washed with TBST for three times and incubated with HRP-conjugated secondary antibody at room temperature for 1 h. Following three times wash with TBST, the membranes were developed by the ECL detection Kit. The images of western blot were captured and analyzed by Bio-rad imaging system.

### Immunofluorescence of Nrf2

Cells were grown on glass coverslips, and after drug treatment, cells were fixed in 4% paraformaldehyde in PBS for 20 min. After washing twice with ice cold PBS, the cells were incubated for 10 min with PBS containing 0.25% Triton X-100. After being blocked with 5% BSA for 30 min, cells were incubated with primary antibody in 0.5% BSA/PBST in a humidified chamber overnight at 4°C. Then washed with TBST for three times and incubated with FITC-conjugated secondary antibody at room temperature for 1 h. Then cells were incubated with 10 μg/mL DAPI and incubated for another 30 min. Signals were visualized and recorded using a Confocal Microscopy (Leica TCS SP8) at a magnification of 400 ×.

### The analysis of drug accumulation ability

Accumulation of Rh123 was determined by incubating cells with Rh123 (2 μM) for 1 h at 37°C. Cells were then placed in ice-water bath and followed by harvesting and washing twice with ice-cold PBS. The fluorescence intensity was measured to determine the intracellular drug accumulation.

### Determination of glutathione (GSH)

After the cell collection, the medium was removed and the cells were washed thrice with PBS. Cells were dissociated by cell lysis buffer, and cell lysis was carried out at 4°C by vigorous shaking for 45 min. After centrifugation at 12000 rpm for 10 min, supernatant was separated and used to measure the GSH content using assay kits based on the specified manufacturer’s instructions.

### Evaluation of drug interactions

The median effect analysis of Chou, which is based on the median-effect principle, was used to calculate synergism or antagonism for the combination index (CI) in drug combination [30]. Using the median-effect equation and the CI equation and plot, concentration effect curves of AA-6 (2), adriamycin and their combination in serially diluted concentrations were plotted. CI values in different concentration and isobolograms were generated using the computer software Excel. In this method, CI values of 1, < 1, and > 1, respectively represent for additive, synergistic, or antagonistic effects [31].

### Homology modeling

The human P-gp protein sequence (Uniprot ID: P08183) was obtained from the UniProt database [32]. The BLAST Search protocol implemented in Discovery Studio 3.1 (Accelrys Inc., San Diego, CA) was used to perform the sequence similarity search and to find the templates for homology modeling. We then selected the X-ray crystal structure of murine P-gp (4M2S.pdb [33]) with a sequence identity of 87% as the template for constructing the homology models of human P-gp. Using this template, five homology models of human P-gp were generated using the Macromolecules module in Discovery Studio 3.1. Based on the DOPE (discrete optimized protein energy) score, the homology model M002 was selected for the docking studies.

### Docking

Predicted bound configurations for compound AA-6 (2) were obtained using GOLD (version 5.0 [16]) with the homology model M002 representing the human P-gp structure. The preparation of the protein structure including adding hydrogen atoms was conducted using the wizard in GOLD. The binding position of the ligand QZ59-RRR in 4M2S.pdb [33] was used as the reference to define the ligand binding site. The three-dimensional structure of compound AA-6 (2) was constructed and minimized using ChemBio3D ultra (version 12.0, CambridgeSoft Corporation, USA). The default settings for the genetic algorithm in GOLD and the GoldScore function were used to dock the ligand and rank the ligand poses. The docking pose of compound AA-6 (2) was visualized using PyMOL (version 0.99rc6, Schrödinger Inc., New York, NY, USA).

### P-gp Substrate Prediction

We used Supek’s models based on the algorithms of support vector machine and decision tree to predict whether AA-6 (2) is a P-gp substrate [18]. The prediction models were provided by Supek and co-workers as a web application, which can be accessed by visiting http://pgp.biozyne.com/. The input we used is the SMILES string of AA-6 (2), CC(=O)OC(CC1)C(C)(C)C(CC2)C1(C)C3CCC(C4C23C) C(CC4)C(C)(O)CCCC(C)(C)OO.

### Statistical analysis

All the experiments were performed in triplicates. All data were presented as means (± S.D.). Significant differences between the groups were determined by One-way ANOVA followed by Dunnett’s multiple comparison tests. *P*-values less than 0.05 were considered as statistically significant. Typical results from 3 independent experiments were chosen and shown in Figure 2A, Figure 2D, Figure 3A–3C, Figure 4C, and Figure 4D.

## SUPPLEMENTARY MATERIALS FIGURES


