# Supplementary material for: Triterpenoids from Aglaia abbreviata exert cytotoxicity and multidrug resistant reversal effect in MCF-7/ADM cells via reactive oxygen species induction and P-glycoprotein inhibition
Source: Oncotarget. 2017 Apr 20;8(41):69465–76. doi: 10.18632/oncotarget.17287 (PMC5642492; doi:10.18632/oncotarget.17287)
Supplement: Supplementary file 1 [file oncotarget-08-69465-s001.pdf]

Triterpenoids from *Aglaia abbreviata* exert cytotoxicity and multidrug resistant reversal effect in MCF-7/ADM cells via reactive oxygen species induction and P-glycoprotein inhibition

Supplementary Materials

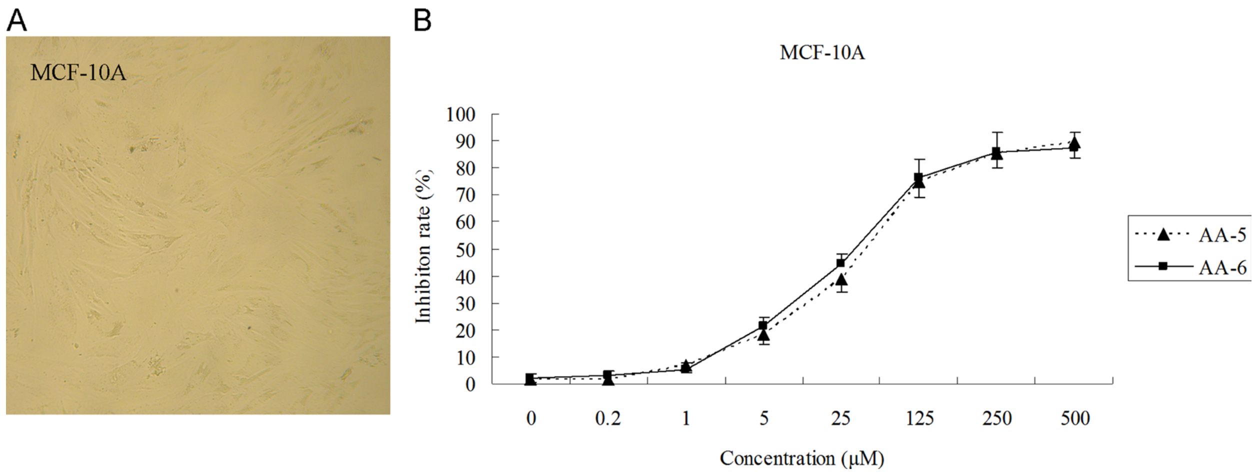

Supplementary Figure 1: AA-5 (1) and AA-6 (2) showed weak inhibition of cell viability in MCF-10A cells. (**A**) The cytological morphology of MCF-10A cells. (**B**) AA-5 (1) and AA-6 (2) inhibited growth of MCF-10A cells in a dose-dependent manner by MTT assay. Cells were treated with same volume of DMSO, 0.2, 1, 5, 25, 125, 250, 500 μM AA-5 (1), AA-6 (2) on MCF-10A cells for 48 h. Data represents the mean ± SD, $n = 3$. The IC50 of AA-5 and AA-6 in MCF-10A cells was 24.52 and 21.4 μM, respectively.

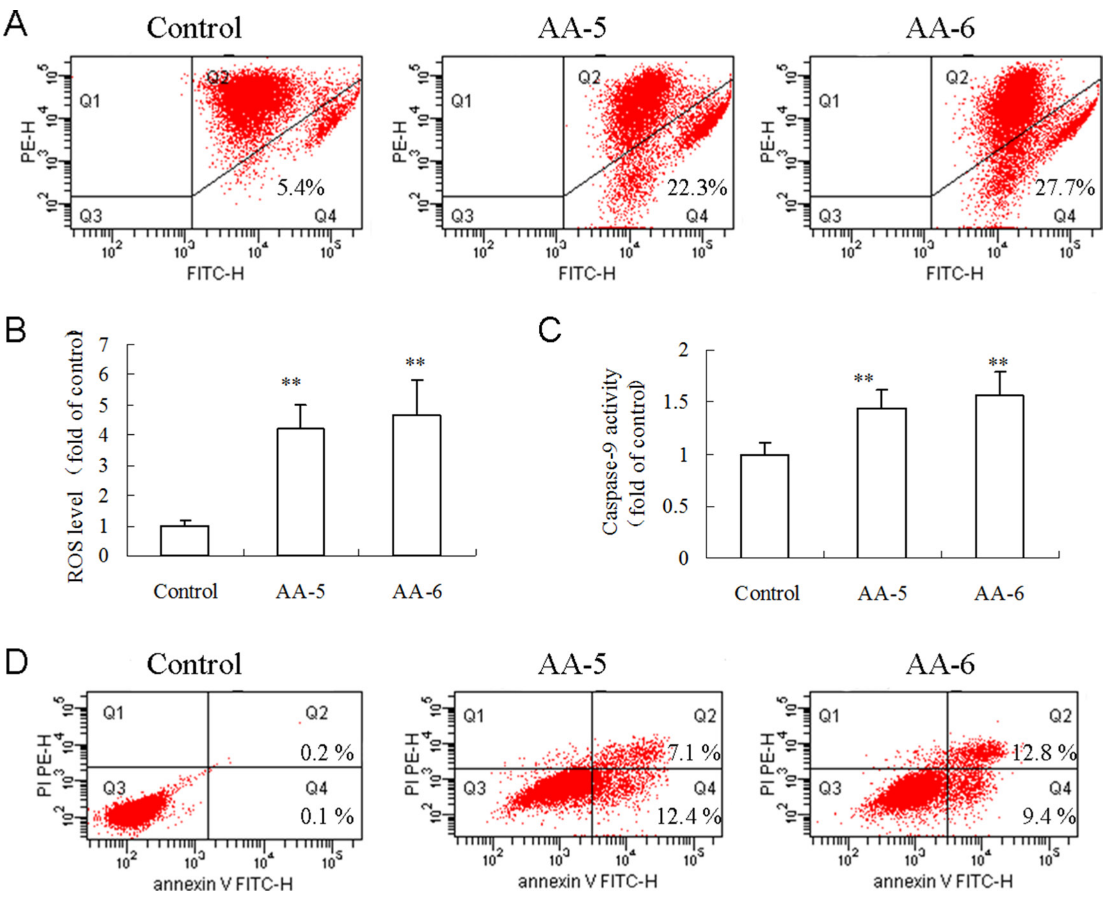

Supplementary Figure 2: AA-5 (1) and AA-6 (2) induced mitochondria-mediated apoptosis in MCF-7 cells. (**A**) Depolarization of mitochondria membrane potential in response to AAs treatment. (**B**) Reactive oxygen species generation in response to AAs treatment. (**C**) Activation of caspase-9 induced by AAs treatment. Data represents the mean ± SD, $n = 3$, significant differences relative to control were indicated as $^{**}P < 0.01$. (**D**) Flow cytometric analysis of AAs induced apoptosis in MCF-7 cells. Cells were incubated with same volume of DMSO, 5 μM AA-5 (1), or AA-6 (2) for 48 h.

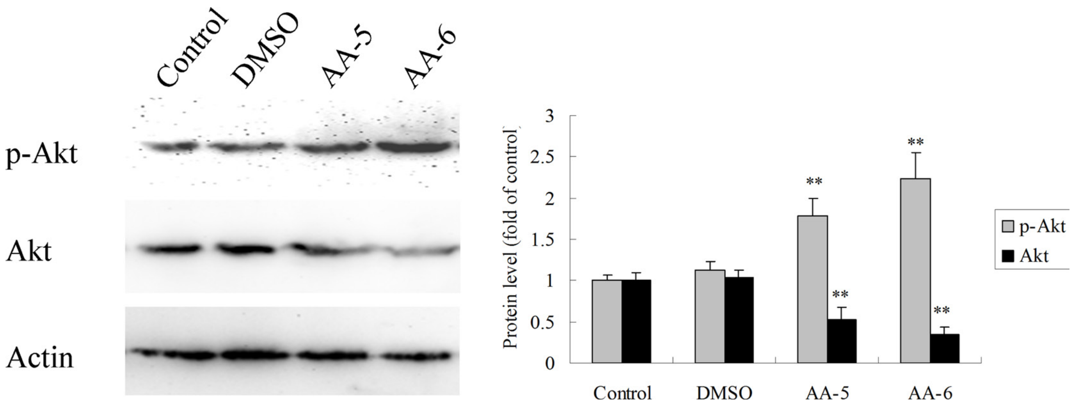

Supplementary Figure 3: Short-term treatment of AA-5 (1) and AA-6 (2) increased the activity of Akt signal in MCF-7/ADM cells. Cells were respectively treated with same volume of DMSO, 10 μM AA-5 (1), and 5μM AA-6 (2) for 2 h. Data represents the mean ± SD, $n = 3$, significant differences relative to control were indicated as $^{**}P < 0.01$.

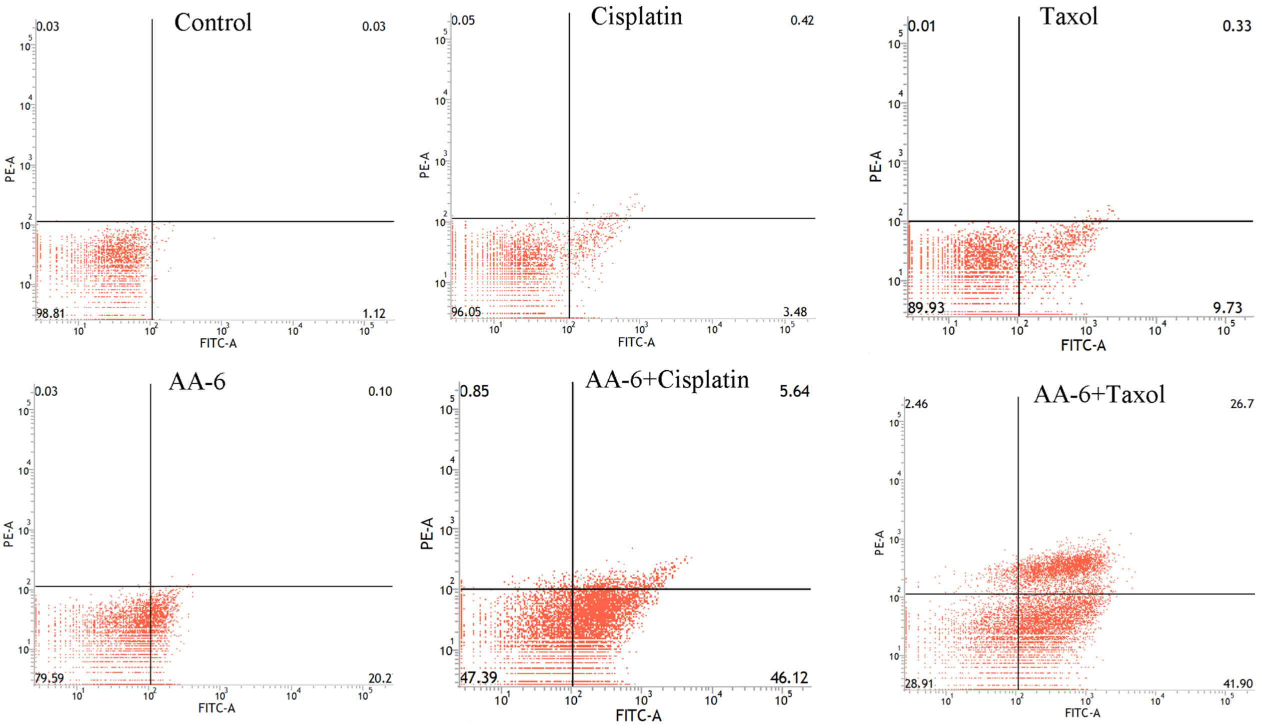

Supplementary Figure 4: Flow cytometric analysis of cisplatin and taxol induced apoptosis with or without AA-6 (2) in MCF-7/ADM cells. Cells were incubated with 1 µM cisplatin or taxol with or without 1 µM AA-6 (2) for 48 h. Cells in control group were treated with same volume of DMSO.
